# Foundations of Neuroeconomics: From Philosophy to Practice

**DOI:** 10.1371/journal.pbio.0060298

**Published:** 2008-11-25

**Authors:** John A Clithero, Dharol Tankersley, Scott A Huettel

**Affiliations:** University of Minnesota, United States of America

## Abstract

How can we use neuroscience to better understand economic behavior? By quelling concerns about the nascent field of neuroeconomics, the authors defend future integrations of the biological and social sciences.

Evidence that neuroscience improves our understanding of economic phenomena [1–4] comes from a broad array of novel experimental findings, including demonstrations of brain regions that guide responses to fair [5,6] and unfair [7] social interactions, that resolve uncertainty during decision making [8], that track loss aversion [9] and subjective value [10], and that encode willingness to pay [11,12] and reward error signals [13,14]. Yet, neuroeconomics has been characterized as a faddish juxtaposition, not an integration, of disparate domains [15]. More damningly, critics have charged that neuroscience and economics are fundamentally incompatible [16], an argument that resonates with many social scientists. Economics thrived for centuries in the absence of neuroscience and some economists argue that existing neuroeconomics research is not useful to mainstream economics [17,18].

We reject the fundamental charge that neuroscience cannot influence economic modeling, even in principle, and focus on two criticisms of integrating these fields, which we label the *Behavioral Sufficiency* and *Emergent Phenomenon* arguments. We show here that these arguments contain hidden assumptions that render them unsound within the practical constraints of science.

We go on to explore two interrelated questions: is there a unique niche for a field of neuroeconomics, and, if so, what are its proper foundational principles? We do not rely on the recent demonstrations of brain systems that support economic behavior nor recount the valid concerns about potential technological constraints of neuroscience. Rather, we attempt to clarify the necessary foundations for neuroeconomics research [19–21], for which we identify two core principles, *Mechanistic Convergence* and *Biological Plausibility*.

We then ask how information about neural mechanisms improves the predictive and explanatory power of economic models. Importantly, the points we raise here recapitulate both the cognitive revolution [22] and the subsequent intertwining of cognitive psychology and cognitive neuroscience [23,24]. We believe that the seemingly disparate neural and social sciences have much to gain from each other.

## Neuroeconomics: Promise Unfulfilled?

We define neuroeconomics as the convergence of the neural and social sciences, applied to the understanding and prediction of decisions about rewards [25], such as money, food, information acquisition, physical pleasure or pain, and social interactions. Neuroscience brings a wealth of technological approaches, including brain imaging [e.g., functional magnetic resonance imaging (fMRI)], lesion studies, molecular biology, pharmacology, and electrophysiology. Economics adds conceptual principles (e.g., rationality and utility), statistical techniques, and rigorous modeling. Psychology provides evidence for decision biases such as heuristics, framing effects, and emotional influences. Finally, genetics [26,27], computer science [28], and philosophy [21,29,30] contribute to neuroeconomic research. Numerous reviews summarize research at the intersection of these fields [1,3,31–35].

Among neuroscientists, the incorporation of economic concepts has generated much excitement. Economic models make assumptions about “covert preferences” [36], or value judgments, because measuring actual preferences with only behavioral methods is difficult [37]. But neuroscience may provide a means to measure those covert preferences [18], potentially eliminating the need for those assumptions. More broadly, neuroscience often incorporates economic models to explain brain function, both when investigating decision making under risky conditions [38–40] and examining information acquisition during learning [41,42]. Based on the breadth of research so far, introducing economic concepts has led to clear advances within social and decision neuroscience.

Nonetheless, detractors dispute the value of neuroeconomics, often citing poor statistical practice [17], the answering of irrelevant questions [16], skepticism about the relevance of nonhuman animal studies [16], and the interpretational difficulties associated with neuroimaging data [43]. Whereas neuroscience data is compelling because it seems to reveal previously inaccessible truths [44,45], the lack of functional specificity of many brain regions (at least at the level accessible to common neuroimaging techniques) often precludes strong conclusions about links between brain regions and behavior [46]. Even where neuroscience can ask well-formed questions, the economic literature may have different disciplinary conventions (e.g., statistical analyses and decision models) that preclude ready translation between the fields [17,18]. Without a common language or principles to bridge the disciplines [19,20], neuroeconomics may become increasingly brain-centric.

Moreover, neuroscientists and social scientists work with different methods and datasets. Neuroscientists frequently require expensive hardware, use invasive techniques, and draw data from a small sample of humans or animals. Social scientists, in contrast, generally measure information about choice preferences (or other forms of behavior) through relatively inexpensive laboratory testing, and often use data from observations in natural environments (e.g., housing prices), across large and diverse samples of subjects. These many disciplinary contrasts have led critics to make two arguments, which we here label *Behavioral Sufficiency* and *Emergent Phenomenon*, that neuroscience data cannot influence economic modeling, even in principle.

## Arguments Against Neuroeconomics

### Behavioral Sufficiency.

Some theorists argue that economic hypotheses cannot be falsified using neuroscience data [16,47]. Since economic models make no assumptions about the mechanisms underlying behavior, the argument goes, no data about those mechanisms could confirm or refute any economic model [16]. To falsify an economic model, researchers must manipulate some environmental factor and observe a change in behavior contrary to the model's predictions. In this *Behavioral Sufficiency* argument, behavioral data are both necessary and sufficient to evaluate the validity of economic models, leaving only brain function or clinical disorders for neuroeconomics to address.

This argument builds from the concept of revealed preference in economics [48,49]. Economic models emphasize observable choice data to construct sets of preferences sufficient to model and predict choice [21]. For example, consider the hypothesis that affective feelings (e.g., sadness) exert an effect on decisions [50]. Although this hypothesis is a natural candidate for models inspired by neuroscience, in that it posits specific intervening states that influence choice, at present identifying those states requires self-reported behavioral data (e.g., feelings of sadness), not neuroscience proxies (e.g., amygdala activation). Even if nothing were known about the neural mechanisms of emotion, choice, or their interaction, behavioral research could reveal that sadness biases decisions [51]. Models can incorporate other affective states (e.g., anger or fear) by inducing feelings and then testing the consequences on decisions. In all cases, purely behavioral data (the subjects' responses to induced states) would be sufficient, in principle, to identify relationships between independent variables associated with an environmental manipulation and a dependent variable associated with a real-world decision measure. Thus, critics argue, behavioral models, not mechanism-based models, can facilitate prediction.

Researchers can collect behavioral data from hundreds of individuals at relatively little cost. For many economic questions, data from laboratory experiments merge with observations of real-world behavior, providing important checks on the validity of research phenomena. In contrast, neuroscience experiments require large-scale capital investments and specialized skills for data collection and analyses, and necessarily constrain participants' behavior dramatically: body movements, face-to-face interactions, and verbal expressions of decisions are all restricted. The small sample size of neuroscience experiments complicates analyses of individual differences, and even well-conducted, adequately powered experiments may lead to equivocal conclusions, because of inherent limitations in the experimental methods [43,52] and incomplete knowledge about underlying brain function [46]. In short, given the challenges of neuroscience experimentation compared to traditional social science methods, the *Behavioral Sufficiency* argument seems to sound the death knell for neuroeconomics.

Yet, the simplicity of this argument belies a hidden premise that undercuts its practical validity. We do not disagree that researchers could falsify any possible economic model using behavioral data and may predict behavioral phenomena without an understanding of mechanism. However, *Behavioral Sufficiency* rests on the premise that the data necessary to falsify or support a model can, in practice, be identified and collected. Many important decision phenomena require a wide range of tests for their experimentation. For the hypothesis described earlier [50], researchers could test effects of a whole range of affective states, such as anger, depression, elation, and sleep deprivation. With comprehensive data about how preferences change across these states, theorists could validate, reject, or refine relevant economic models, but accumulating those data would be time-consuming and expensive, especially when testing interacting factors (e.g., the effects of depression in adolescents upon delay discounting). Thus, the argument succeeds in principle but fails in practice: no researcher—indeed, no collection of researchers—can obtain all possible data about all possible behaviors.

These practical limitations leave an opening for neuroscience data to influence economic modeling by directing the course of research. Continuing the above example, long-standing neuroscience work distinguishes emotional states as resulting from different mechanisms, with disgust, pain, and fear, for example, all reflecting different neural substrates [53,54], despite some superficial similarities. So, neuroscience data that map particular affective systems (e.g., posterior rather than anterior insula) to specific forms of choice (e.g., purchasing decisions) may suggest new directions for subsequent behavioral research [12]. We believe that neuroscience could make important contributions to economics by improving the efficacy (both in falsification and explanation) of behavioral research.

### Emergent Phenomenon.

A second criticism focuses on the methods of neuroeconomics, which frequently place human (or monkey or rodent) subjects in a mock economic setting to elicit a desired behavior—such as differential framing of gains and losses [9,55], rejection of unfair offers [7], or incentivized memory retention [56]—and identify its neural correlates. Neuroscientists make similar extrapolations from animal studies when monkeys make decisions regarding juice [10] or rats make decisions involving drops of sucrose [57]. In effect, these studies create a simplified “toy model” of a real-world phenomenon in order to test hypotheses about an underlying mechanism. Toy models have long been used in both the natural sciences (e.g., placing small-scale structures into wind tunnels to understand fluid mechanics) and the biological sciences (e.g., in vitro studies to understand cellular properties), but economists have only relatively recently started to use them, creating simple markets (or other economic institutions) within the laboratory [58,59] and inducing subjects to behave in a self-interested manner reflective of real-world behavior. These markets obeyed basic research principles: participants received full and accurate information, decisions had meaningful (usually monetary) consequences, and deception was prohibited. Neuroeconomic experiments typically follow these principles. In recent studies of purchasing decisions, for example, subjects had the opportunity to spend real money and receive real goods (e.g., iPods and candy) in return [11,12]. Researchers extrapolate that the neural mechanisms recruited in such laboratory studies also underlie real-world purchasing behavior.

The validity of a toy model rests on the assumption that the principles of interest are maintained from the laboratory setting to the natural environment. When the physical principles change—as when moving from small-scale models of buildings to their real-world counterparts—an extrapolation from toy models may have disastrous consequences [60]. Neuroeconomic experiments can suffer from similar problems: principles that shape behavior in the laboratory (e.g., experimenter demand effects) do not necessarily influence real-world phenomena (e.g., amount of charitable giving). Suppose that an economist and neuroscientist create a mock retirement-planning fMRI experiment to identify ways to encourage participation in a retirement savings plan and discover patterns of neural activation that predict decisions to save money. Critics charge that because information about neural mechanisms was collected from a few dozen subjects, it cannot generate a better understanding of retirement planning behavior for millions of adults. In this argument, the underlying mechanisms have no bearing on economic theories that describe aggregate data. Similar and notable claims have been made within economics about market phenomena [61]. We refer to this as the *Emergent Phenomenon* argument: the denial that an understanding of mechanism has relevance for understanding phenomena at the aggregate societal level. Similar reasoning pervades criticisms of behavioral economics. Consistent with substantial evidence that emergence is common within complex economic systems [62,63], this argument limits the scope of neuroscience data from generalizing to higher-level phenomena.

The *Emergent Phenomenon* argument, however, rests on the assumption that emergence not only exists but subsumes any influences from lower levels. Yet, as has been argued with respect to macroeconomic modeling [64], emergence may not be ubiquitous among economic phenomena. Microeconomic theory invokes the concept of a general equilibrium [65] to explain aggregate market outcomes based on individual behavior and preferences, a foundational concept that explains higher-level outcomes from lower-level data. Consider drug addiction, a social problem of increasing interest to economists [66,67]. No scientist claims that understanding the neural mechanisms of addiction provides a complete explanation of drug abuse, but that understanding undeniably clarifies both the etiology of addiction, as evident in genetic influences [68] and the success of interventions like nicotine patches, leading to clear changes in public policy. Other economic phenomena may have only limited emergence. For example, retirement planning in older adults is likely influenced by general cognitive decline with aging [69], and the financial decisions of teenagers reveal a broad pattern of impulsivity, which reflects delayed neural development of the prefrontal cortex compared to other brain systems [70]. That some economic phenomena have some emergent properties restricts the explanatory power of hierarchical, mechanistic models, but does not render those models logically invalid.

Social science models are now increasingly likely to incorporate some mechanistic explanations that account for effects across levels. As an example, economic experiments aimed at implementing general equilibrium theory in the laboratory use individual portfolio choices to explain financial market behavior [71]. To the extent that researchers can more accurately specify the mechanisms underlying the behavior of an individual, some phenomena of interest to economists will be better modeled. A core goal of neuroeconomics will be identifying those economic phenomena to which neuroscience can be most profitably applied.

## Foundations of Neuroeconomics

### Mechanistic Convergence in experiments.

Behavioral economic research [72,73] could proceed without neuroscience, but we believe that neuroscience data will increase the efficiency of this research. Specifically, via *Mechanistic Convergence*, neuroscience experiments can guide the generation and direction of future behavioral studies with a multi-stage “behavior-to-brain-to-behavior” approach.

By identifying interesting choice behavior and creating models for the associated cognitive processes, neuroeconomics research can generate better paradigms for human neuroimaging studies and target behavior to replicate in animal and clinical studies (*behavior*). By grounding conclusions about brain function in behavioral effects such as choice parameters or individual decisions, neuroeconomics can unify cognitive and neural theories of behavior [18]. Knowledge about the underlying mechanisms (*brain*) can generate novel hypotheses about external modulating factors, which in turn can be generalized to new samples, tasks, and experimental environments (*behavior*). Well-designed neuroscience experiments can speed the course of behavioral research, effectively using mechanistic knowledge to target observable behaviors for subsequent experimentation. This use of convergent evidence from neuroscience refines and reduces the number of experiments necessary for understanding individual behavior.

Neural and behavioral studies should interact to identify interesting phenomena, to suggest mechanisms that underlie those phenomena, and to map out the biological substrates that support those mechanisms [18]. This iterative approach is also important when multiple decision or psychological processes could lead to the same choice behavior [20]. For example, temptation (e.g., purchasing a sale item because it is a good deal) and regret (e.g., purchasing a sale item to avoid future regret) are different subjective phenomena but can lead to similar purchasing decisions [74], despite likely having distinct neural substrates [75]. Neuroscience data that distinguish these affective states can guide the construction of new behavioral experiments and more-targeted hypotheses. Such data could differentiate properties of regret and temptation, along with related phenomena that may share some of their neural mechanisms, increasing the efficiency of behavioral research.

### *Biological Plausibility* in models.

Aside from producing new hypotheses, neurobiological knowledge can also introduce constraints. Models of neural function have guided theories of executive control and decision making [76–82]. Likewise, integrating psychological concepts into models is not new to economics [83,84]. We argue that neuroscience can inspire models of behavior that conform to our current scientific knowledge, i.e., behavioral models that have *Biological Plausibility.* The advantages of mechanistic knowledge are well documented in the psychological, philosophical, and economic literatures [19,85]. For example, a combination of rodent [57,86], nonhuman primate [87,88], and human studies [41,42,89] have led to theorizing about the role of dopamine in reward processing and prediction error. To the extent that neuroeconomics provides insight into the mechanisms guiding different forms of utility, such knowledge constrains candidate models of individuals' choice processing.

Yet, only a handful of microeconomic models strive for biological plausibility. The dual-systems framework postulates that choice reflects the interaction of two distinct neurocognitive systems with complementary strengths and weakness [83]. One common dichotomy separates automatic or “hot” affective processes from controlled or “cold” cognitive processes, and similar divisions are used in several economic models [67,90–93]. Critically, the dual-systems models are compelling because they are intuitively plausible and supported by both human [55,94] and animal [95,96] neuroscience data. These models generate testable hypotheses about the nature and timing of interactions between competing brain systems, which may, of course, lead to the rejection of these models. For example, recent studies suggest that a more unified set of neural processes support the evaluation of options and decisions [9,97,98]. Constraining theories in accordance with our best neurobiological knowledge is critical for moving beyond behavioral conflations of several distinct affective states. Full understanding of the mechanisms of choice will require more precise characterization of precipitating, modulating, and inhibiting factors [85], potentially through neuroscience data.

We note that most current neuroscientific methods provide only coarse information about mechanism. The dominant technique, fMRI, provides temporal information about the relative metabolic demand of populations of hundreds of thousands of neurons [43]. Models based on these methods must make simplifying assumptions: e.g., region *A* activates as if it is modulated by region *B*. Note that this simplification is similar to the aforementioned “revealed preference” foundation of economic choice models: the actual mechanistic relation between regions *A* and *B* is not being modeled, just as the actual computations underlying preferences are generally ignored. New methods in fMRI, notably those that characterize how information shapes connections between regions [99], promise to create integrated models that include both a description of information flow among brain regions and the effects of behavior upon the connections between those regions. Integrating techniques within single studies, such as fMRI and genetics [27,100], will be critical for producing mechanistically complete and biologically plausible explanations of behaviors.

## Conclusion

Neuroeconomics is at a crossroads, poised to demonstrate that neuroscience can provide the same types of benefits it has long received from the social sciences. Ideas from game theory and expected utility theory can explain the responses of individual neurons to incoming information [2]. Similarly, aspects of utility theory can be used to describe the activity of populations of neurons within the brain's reward system [101]. There is also an opportunity for the axiomatic approach of decision theory to explain decision-making mechanisms [20], such as building from the response properties of dopaminergic neurons [102]. Without comparable examples of neuroscience data contributing to economic models, critics could argue that neuroeconomics research is a brain-centric enterprise that incorporates ideas from the social sciences without reciprocation [16,17].

We agree that neuroeconomic research has indeed been brain-centric, but stress that it need not remain so. The core criticisms of neuroeconomics constrain the practice of this field, but do not render it meaningless. Clear foundational principles remain. Neuroeconomics via *Mechanistic Convergence* can more efficiently direct the course of future behavioral studies. As an historical parallel, economists have explored psychological concepts (e.g., emotional influences on decision making) for many years [84,103–105], sparking a broad array of new behavioral experiments and theories. Furthermore, neuroeconomics can facilitate the creation and testing of models that adhere to *Biological Plausibility*. Social scientists (and neuroscientists) should not treat decision-making phenomena as irreducible and mechanism-independent. Instead, the joint investigation of brain and behavior will lead to greater success than either discipline could achieve in isolation.

## Abbreviations

fMRI: functional magnetic resonance imaging
